# Participatory testing and reporting in an environmental-justice community of Worcester, Massachusetts: a pilot project

**DOI:** 10.1186/1476-069X-9-34

**Published:** 2010-07-06

**Authors:** Timothy J Downs, Laurie Ross, Danielle Mucciarone, Maria-Camila Calvache, Octavia Taylor, Robert Goble

**Affiliations:** 1 Environmental Science and Policy Program, Clark University, 950 Main Street, Worcester, Massachusetts 01610, USA; 2 George Perkins Marsh Research Institute, Clark University, 950 Main Street, Worcester, Massachusetts 01610, USA; 3 Community Development and Planning Program, Clark University, 950 Main Street, Worcester, Massachusetts 01610, USA

## Abstract

**Background:**

Despite indoor home environments being where people spend most time, involving residents in testing those environments has been very limited, especially in marginalized communities. We piloted *participatory testing and reporting *that combined relatively simple tests with actionable reporting to empower residents in Main South/Piedmont neighborhoods of Worcester, Massachusetts. We answered: 1) How do we design and implement the approach for neighborhood and household environments using participatory methods? 2) What do pilot tests reveal? 3) How does our experience inform testing practice?

**Methods:**

The approach was designed and implemented with community partners using community-based participatory research. Residents and researchers tested fourteen homes for: lead in dust indoors, soil outdoors, paint indoors and drinking water; radon in basement air; PM2.5 in indoor air; mold spores in indoor/outdoor air; and drinking water quality. Monitoring of neighborhood particulates by residents and researchers used real-time data to stimulate dialogue.

**Results:**

Given the newness of our partnership and unforeseen conflicts, we achieved moderate-high success overall based on process and outcome criteria: methods, test results, reporting, lessons learned. The conflict burden we experienced may be attributable less to generic university-community differences in interests/culture, and more to territoriality and interpersonal issues. Lead-in-paint touch-swab results were poor proxies for lead-in-dust. Of eight units tested in summer, three had very high lead-in-dust (>1000 *μ*g/ft^2^), six exceeded at least one USEPA standard for lead-in-dust and/or soil. Tap water tests showed no significant exposures. Monitoring of neighborhood particulates raised awareness of environmental health risks, especially asthma.

**Conclusions:**

Timely reporting back home-toxics' results to residents is ethical but it must be empowering. Future work should fund the active participation of a few motivated residents as representatives of the target population. Although difficult and demanding in time and effort, the approach can educate residents and inform exposure assessment. It should be considered as a core ingredient of comprehensive household toxics' testing, and has potential to improve participant retention and the overall positive impact of long-term environmental health research efforts.

## Background

Urban residents in North America typically spend over 90 percent of their time indoors [1]. While the monitoring and regulation of hazardous air pollutants has focused on outdoor environments [2], exposures indoors may be more important because of lower dilution rates and higher proximity to sources; for example inhalation rates for environmental tobacco smoke may be 100 times higher indoors vs, outdoors [3,4]. Privacy and private-property restrictions necessarily limit the extent to which governmental agencies can regulate the home environment. A notable exception was precipitated by the ban placed on lead in domestic paint by the U.S. Consumer Products Safety Commission on January 1^st^, 1978: some states, like Massachusetts, require the removal or covering of lead paint hazards in homes built before 1978 where any children under six live, and mandates testing of blood-lead levels [5].

Our goal is to report on *participatory testing and reporting *(PT&R) - an approach to environmental testing that enables the inhabitants of those environments to participate in meaningful and empowering ways in the testing activity, and reports-back actionable results in a timely fashion. Three research questions were foci: 1) How do we design and implement PT&R for neighborhood and household environments using community-based participatory research (CBPR)? 2) What do pilot tests reveal? 3) How does our experience inform testing practice? Environmental testing had two goals: a) to pilot methods that could be used for an expanded PT&R program that builds capacity to undertake low-cost, easily implementable community-based monitoring and raises awareness among vulnerable groups; and b) to gain insight into the levels of chosen indicators in pilot homes and whether they provide useful exposure information. Detailed quantitative characterization of the home and outdoor environments of the neighborhood was not an aim.

PT&R was one part of a holistic environmental justice/community-based participatory research (EJ/CBPR) parent project called: "Strengthening vulnerable communities in the Worcester built environment - Neighborhood STRENGTH" (2004-2008). STRENGTH had four partners: a community center dedicated to youth development; an environmental outreach non-profit; a community based health center; and Clark University [6]. Its five parts were: 1) PT&R of indoor and outdoor pollution; 2) learning about residents' health needs and concerns through community-based listening sessions; 3) collaborative survey work, including a household vulnerability survey and an asthma prevalence survey for schoolchildren (focus of [7]); 4) tackling persistent street trash and illegal dumping strategically; and 5) educating and empowering youth to promote environmental justice. Each was chosen by the partnership because it was: a) a high community concern (expressed in pre-proposal focus groups with residents, by our community partners, and in previous studies); and b) there had been insufficient attention paid to it to date.

Community-based participatory research draws on participatory models [8-10] and promotes active involvement by communities in the shaping and conduct of research and intervention [11]. Understanding environmental exposures to risk agents using CBPR is challenging: Power and privilege inequities among partners; racial and ethnic discrimination; reconciling academic and advocacy/activism cultural differences; and how best to use research for social change are among tensions that impact partnerships [8,12-14]. Continuous conflict resolution and negotiation are needed to address them [15]. The particular challenges we faced are described in Results, and compared with other work in Discussion.

Household-level environmental testing and the use of participatory methods are both topical. The *Healthy Homes Project *(HHP) has taken a comprehensive approach to housing-related hazards research [16]. One study estimated that 38 million housing units in the U.S had lead-based paint, 24 million with significant lead-based paint hazards: greatest health risks occur in older units occupied by low-income families with children less than six years of age [17]. Another HHP study shows poor housing conditions to be associated with a wide range of health conditions, including respiratory infections, asthma, lead poisoning, injuries, and mental health. It argues that public health departments should employ multiple strategies to improve housing, such as developing and enforcing housing guidelines and codes [18].

Asthma has been a HHP focus. Krieger et al. [19] assessed community health worker (CHW) interventions designed to reduce exposure to indoor asthma triggers in Seattle-King County. Community members trained as CHWs collected dust samples (tested for allergens) and undertook visual inspections and interviews. Working together CHWs and participants prioritized interventions based on test results. In similar asthma work in Baltimore, home environments were tested for NO_2_, O_3_, airborne particulates, and allergens pre- and post-intervention [20]. Alameda County Lead Poisoning Prevention Program is working to improve the lives of asthmatic children by providing multi-hazard housing interventions (including allergen reduction, moisture and ventilation control) and in-home education [21].

PT&R builds on a firm foundation of community-based environmental health research that took-off in the 1990s-2000s. The *Healthy Public Housing Initiative *(HPHI) is an ongoing effort to understand and improve the health of Boston public-housing residents, especially children with asthma [22]; it trains public housing residents to conduct community-based surveys of their neighbors. HPHI started in 1998 with a participatory 150-question survey of health and housing conditions in the West Broadway Housing Development. Follow-up pilot interventions with nine families identified the most severe indoor environmental threats to asthmatic children [23]. Working in Boston's Roxbury district since 1994, *Alternatives for Community and Environment *(ACE) focuses on issues of youth, air pollution, transportation, and environmental health [24]. Well established, ACE blends research rigor, community building, and policy advocacy, and has a multi-cultural workforce that reflects its target community - attributes that facilitate PT&R. In our pilot, we were interested in what a more typical community-university partnership could achieve with residents.

Another foundational effort that informs our PT&R work is the USEPA's *Community Action for a Renewed Environment *(CARE) Program which has funded community-based research to understand exposure to toxic substances in homes nationwide; since 2005 it has touched 50 communities in 26 states. In Tucson, Arizona, community outreach workers completed environmental health trainings, about 700 home visits and 1,400 screening tests for lead [25]. In 2006, the International District Housing Alliance (CARE project in Seattle, WA) held focus group meetings with residents who prioritized indoor and outdoor air quality issues, and this led to indoor air workshops and in-home assessments for 95 residents [26]. CBPR applied to pollution, human health and community health has become more common [27]. While PT&R-type methods are not mentioned specifically, they are wholly consistent with this approach.

Also of relevance is the *Protocol for Assessing Community Excellence in Environmental Health *(PACE EH), an approach to conducting *community environmental health assessments *(CEHAs) using CBPR in the US and abroad [28]. PACE pays attention to survey methods, data collection and analysis, outreach and communication. For example, in Cincinnati OH, CBPR helped families identify and reduce health risks from lead, pesticides and, other environmental hazards. Working with technicians, 130 participating families collected one floor dust wipe for lead, and one floor dust wipe for pesticides. The study concluded that families can adequately screen their housing units to identify lead hazards [29]. The *Alliance for Healthy Homes *(AHH) is a national nonprofit working to prevent and eliminate hazards in homes, including lead, mold, carbon monoxide, radon, pests, and pesticides. Supportive of a PT&R-type approach, AHH concludes: "Researchers need to reveal conclusions and recommendations in a timely manner.... Early advice about principles and the direction of needed change based on preliminary results is valuable, even if definitive advice and standard setting requires more study" [[30] p.1]. It further concludes: "Hazard evaluation and control tools and terminology are usually designed for worst-case situations and to meet the highest burden of proof. While greater precision and reliability is sometimes needed, simple tools that point to corrective and preventive action are also valuable, given that risks in housing run the gamut from the miniscule to the extreme".

Arguably representing the state-of-the-art in participatory exposure assessment, in 2006, *Communities for a Better Environment *(CBE) and *Silent Spring Institute *(SSI) undertook a household exposure assessment in a Northern California environmental justice community [31]. In follow-up work, the same partners interviewed residents of Richmond, CA, about their health, their family members' health and their neighborhood [31]. In other work, SSI studied household exposures to 89 endocrine disrupting chemicals in air and dust by testing 120 Cape Cod homes [32], and the approach is being replicated elsewhere [33].

While such examples of CBPR approaches to assessing environmental exposures exist in some abundance, the particular emphasis on PT&R's processes and outcomes remains less well documented, especially in marginalized populations. A search of PubMed reveals 270 papers using the key phrase "household environmental testing", but only two when the word "participatory" is added [34]; one is a general CBPR approach to asthma research [35], and the other is our paper on the parent project [6]. Making PT&R the focus here, we sharpen our attention to practical concerns with participatory exposure assessment.

## Methods

### Study Site

Worcester, Massachusetts is the second largest city in New England (2006 population 175,500). In the 19^th ^century, Worcester and the Blackstone River Valley were the birthplace of the U.S. Industrial Revolution, a bustling place of canals, mills and factories. The city has one of the highest minority (people of color) populations (22.9%) and one of the lowest average incomes ($35,000) in Massachusetts, but is also the second most extensively overburdened community by environmental hazards in the state [36]. The Main South and Piedmont neighborhoods of Worcester, are the most densely populated, have the highest rates of minority residents, the lowest income, and the highest crime rates in the city. They are recognized as "vulnerable populations" by US Census [37] and environmental-justice neighborhoods by established criteria [6,7]. Residents are simultaneously vulnerable to physical, social and economic stress: high exposures and low adaptation conspire and can be associated with multiple health consequences. Outdoors, for example, there are several important local sources of particulate matter: Interstate 290 runs on the edge of Main South, and New England's largest Inland Container Port is there too, a locus for trucks and trains 24 hours a day, 7 days a week. In pre-project scoping, residents complained of truck noise, traffic congestion and idling trains.

### Community-based participatory research

We obtained Institutional Review Board approval for the work and informed consent from all research participants. Community based participatory research (CBPR) was used to organize, design and implement the pilot project. The work was "participatory" in two ways: a) academic researchers and community-based groups acted in partnership to conceive, design, initiate and run the project; and b) this partnership engaged with residents who participated in PT&R implementation. Environmental testing for toxics was prioritized by the parent STRENGTH project [6]. Our working group *ToxicsWatch*, comprising 5-6 people, met over a period of three years, on average once a month, defining its mission, priorities, goals and desirable outcomes, then designing, planning and implementing PT&R. Decisions were made after in-depth discussion, and based on consensus. The core participants were the environmental outreach non-profit (two members), a non-profit dedicated to testing the environmental quality of homes (one member), one university environmental health scientist, and one graduate student in environmental science and policy. Researchers thus formed 2/5 of the group. All but one person were residents of Main South. The group's mission became: "To ensure cleaner, healthier and safer places for people to live and work in Main South/Piedmont neighborhoods of Worcester. *ToxicsWatch *promotes environmental health and safety, working to reduce pollution and people's exposure to pollution by: a) the provision of information; b) education and outreach; c) environmental testing indoors and outdoors; and d) organizing for action". ToxicsWatch prioritized two action-oriented activities: 1) neighborhood monitoring of PM10, PM2.5 on local streets; and 2) household-scale environmental testing of indoor air, drinking water, dust, paint and outdoor soil using accessible tests.

### Evaluation

Processes and outcomes were evaluated according to the project proposal, jointly written by the partners. Three *processes *were evaluated: 1) Design and planning of PT&R (for household and neighborhood testing) - post-project, the academics qualitatively and subjectively assessed recruitment of/engagement with residents, and "conflict burden" among partners; unfortunately, residual conflict prevented evaluation of this process by the whole CBPR partnership group. We defined *conflict burden *as the ratio of destructive (i.e. divisive, demoralizing) to constructive conflict among partners - low is desirable, high undesirable [6]; 2) Participatory testing (neighborhood and household) - the partnership group surveyed the ability of residents to actively engage with each of the tests (rated high/moderate/low), and the ability of the residents-partners team to accomplish all the tests within two hours (reasonable threshold of effort); 3) Reporting - the partnership group surveyed residents about useful knowledge gained by them. Within the partnership group, divisive conflicts and mere differences of opinion were differentiated; divisive conflicts tended to divide the group and disrupt a sense of unity - a clash of cultures and personalities that undermined efforts. Outcomes of household PT&R were evaluated by the partnership as follows: 1) testing protocol quality; 2) testing results' utility; 3) reporting quality and information value. Overall experience and lessons learned were assessed using participant observation by the academic researchers and comparisons with other studies.

### Neighborhood particulate matter monitoring

We carried out real-time monitoring of PM2.5 and PM10 with a DustScan Scout 3020 nephelometer (Rupprecht and Patashnick, East Greenbush New York). The small portable device (up to 8-hour battery life) has sufficient sensitivity to assess PM concentrations in clean ambient settings as well as heavily-loaded environments. While some concern exists that the meter tends to overestimate PM levels compared to gravimetric measures [38], it does provide insight on relative concentration patterns. A series of neighborhood walks were held autumn 2005 through autumn 2006, each lasting about two hours, most between 12:00 am-2:00 pm on the weekend. Recruitment by the community-based partner used local outreach: flyers, advertizing, word-of-mouth and phone calls. Participants met, and we shared the goals of the monitoring, the measurement method, and the importance of respirable PM to health. We followed a pre-determined route and a hand-held Garmin GPS60 (Garmin, Olathe, Kansas) was used to track it, synchronized with the Scout's clock. We gathered data in real time as the group of 10-15 people moved along the streets. After the walk, we had a picnic to view PM data. Data were downloaded from the Scout to a laptop computer, and displayed as a graph of PM as a function of time. Later, PM data and GPS data were linked using synchronous time data, and we produced maps of PM levels using ArcGIS 9.3 (ESRI, Redlands, CA) as final output.

### Household Testing

After considerable debate and dialogue over an 18-month period we reached consensus on goals, indicators, and tests. We chose the following eleven tests and wrote a protocol for participatory testing: 1) lead in dust indoors; 2) lead in soil outdoors; 3) lead in paint indoors; 4) lead in drinking water; 5) radon in basement air; 6) PM2.5 in indoor air; 7) mold spores in indoor/outdoor air; 8) drinking water quality - lead, total chlorine, pesticides (atrazine/simazine), total nitrate/nitrite, nitrite, bacteria, pH and hardness; 9) moisture in walls; 10) carbon monoxide sensor status; and 11) visual survey. Methods are summarized in Table 1. We used five criteria for selecting tests: i) the indicator corresponds to a concern expressed by community members in focus groups and community partners on the parent project; ii) the testing for the indicator is affordable for local community groups who would expand the pilot efforts; iii) tests are accessible in terms of being technically simple enough to facilitate participatory testing by target residents; iv) findings from the tests are potentially actionable by residents so that exposures can be reduced; and v) overall the number of tests was sufficient to capture a multi-parameter "healthy homes"-type picture, but not too many to be burdensome. Our approach was in-line with EPA's CARE program guidelines [39]: community concerns included sources of pollution, routes of exposure, and health priorities like asthma and childhood lead poisoning. We considered using an XRF gun for lead testing, but it failed to meet our affordability criterion. We focused primarily on exposure reduction, with data gathering primarily serving that goal; home-hazards characterization was secondary. The testing suite's strength lies in low cost and high accessibility/technical simplicity; its limitation lies in its inability to test for other agents of concern like VOCs/SVOCs.

**Table 1:** Household testing protocol

Indicators	medium	location	materials/methods	MDL^2^	frequency	result type
**1. lead**	**drinking water**	faucet	Water in pipes 6 hours. First-draw sample, then ran the water 60 secs for purged-line sample Used 2 1 L nalgenes. Fill 50 mL small bottles, send to lab (Environmental Quality Institute, Asheville, NC). Lab analysis by EPA Method 200.9, Determination of Trace Elements by Stabilized Temperature Graphite Furnace Atomic Absorption. Rev 2.2, 1994.	1 ppb	once per home	level in ppb

**2. lead**	**indoor paint**	living spaces	LeadCheck^® ^by Hybrivet Systems of Natick, Mass. Touch swabs in six locations, cut beyond surface layer.	1 μg	six locations esp. child contact areas	presence/absence

**3. lead**	**dust**	floors, window sills	Followed Mass. Dept. Env. Protect. Dust Wipe Collection Protocol. Tested three rooms in each home. Dust wipe and a floor wipe in each home. Blank wipe with each home and a spiked wipe in every three homes. Sent to EHS Labs (Richmond VA) for analysis. Analysis by EPA method SW846, 7420.	0.1 μg/ft^2^	7 per home: 3 windowsills 3 floors 1 blank	level in μg/ft^2^

**4. lead**	**outdoor soil**	yard, garden	Followed Worcester Roots protocol. Chose two areas outside to test. Usually a garden and drip line. Took four composite samples in each testing area. Combined the four composites from testing areas #1 into one bag and four composites from testing areas #2 into another bag. Lab analysis by Clark University using ICP-AES3.	1 mg/kg	2 areas per home	level in ppm

**5. radon**	**indoor air**	basement	Used Pro-Lab ^® ^Radon liquid scintillation gas detection canisters. Followed company protocol. Placed canister in basement, away from windows, three feet off the ground. Left for 48+ hours. Sent to Pro-Lab (Weston, FL) for analysis.	0.1 pCi/L	once per home	level in pCi/L

**6. PM2.5**	**indoor air**	living spaces	DustScan Scout 3020 nephelometer (Rupprecht and Patashnick, East Greenbush NY). Optical scattering method.	1 μg/m^3^	Once per room, 30-minute duration	time series, μg/m^3^

**7. suspended mold spores**^1^	**indoor air, outdoor air**	living spaces	CyClex Bioaerosol Impact Sampler, (Environmental Monitoring Systems Inc. Charleston, SC). Rate 20-lpm for 8-10 mins. Impact slide mailed to EMSL Analytical Inc. for analysis of non-viable (non-living) spores1. Analysis by real-time PCR4.	1 count per m^3^	once inside (pilot) once inside and outside (phase II)	Total spore counts per m^3 ^air, ID of spore types

**8. Various**	**drinking water**	kitchen sink, bathroom sink	Parameters (MDL)2: lead (< 15 ppb), total chlorine (< 4 ppm), pesticides (atrazine < 3 ppb, simazine < 4 ppb), total nitrate/nitrite (< 10.0 ppm), nitrite (< 1.0 ppm), bacteria, pH, hardness. WaterSafe^® ^drinking water test (Silver Lake Research Corporation, Monrovia, CA). MDLs are less than USEPA maximum contaminant levels or guideline values as shown.	As shown left in ()	once per home	presence/absence or colorimetric

**9. moisture**	**indoor moisture**	walls, floors	Tramex Moisture Encounter Plus moisture meter (Tramex, Littleton, CO). Wood level on % scale, brick, plaster, drywall on relative scale.	-	variable (0 to 20) per home	% saturation (wood), comparative (plaster etc.)

**10. CO sensor**	**n/a**	indoors	Testing of visibility and operation.	n/a	Once per home	visual and audio detection

**11. visual survey**	**various**	indoors and outdoors	Indicators: deteriorated paint indoors/outdoors; bare soil; cockroaches; rodents; holes in walls; mold/mildew; water damage; strong musty smell; natural gas/sewer gas smell; un-vented gas oven/dryer/heater/stove.	n/a	Once per home	presence/absence


^1 ^includes: *agrocybe, alternaria, ascospore, aspergillus, basidiospores, bipolaris, chaetomium, cladosporium, curvularia, epicoccum, fusarium, ganoderma, myxomycetes, odium, paecilomyces, pithomyces, rusts, scopulariopsis, stachybotrys, taeniolella, torula, ulocladium *and *zygomycetes*.

^2 ^method detection limits; 3 ICP-AES - inductively-coupled plasma-atomic emission spectroscopy; PCR - polymer chain reaction.

Eight homes were tested in pilot phase I in summer 2006, then six others in winter 2007 in phase II. A preparatory training and awareness workshop was held with participants before testing. Phase I homes were chosen from a random sample of 80 households who had indicated willingness to participate during interviews about health and neighborhood life; phase II comprised residents known to the partners and graduate students living in typical triple-decker housing. Thus, the sample was adequate to meet project goals and moderately representative of neighborhood homes: ten tenant-occupied triple-decker units built in the 1930s-40s and four owner-occupied Victorian homes typical of the area. Recruitment was organized by the community-based partner who contacted prospective participants by telephone.

### Reporting

After testing, we interpreted results and tailored reports for each home. The full academic-community-based organization partnership was involved in interpretation of test results and the drafting of reports. The goal was to present residents with a concise, clear report with useful information. The format comprised: Why we test for the pollutant; how to interpret results; what results mean in health terms; and how participants can intervene to reduce exposure. The intervention section emphasized best affordable, accessible options to ensure information was empowering. We held follow-up appointments with residents to discuss results and ways to reduce exposures. Residents in phase I gave us feedback on the household report, including data interpretation, and changes were made for phase II. After reporting, we asked residents to indicate whether overall their PT&R experience had been positive - considering things like what useful knowledge they had gained, effort expended by them to achieve the results, conduct of researchers - and what could be improved. Ongoing work involves simplifying reporting language to a sixth-grade literacy level, and translation to Spanish.

## Results

The evaluation of process and outcome criteria is summarized in Table 2.

**Table 2:** Evaluation results

Criteria process	Household Tests	Neighborhood PM
1. Design and planning of PT&R:		
1a. recruitment of/engagement with residents;	low	low
1b. conflict burden among partners.	high	high

2. Participatory testing:		
2a. ability of residents to engage with tests;	high (10/14), moderate (4/14)	high
2b. ability of the residents-partners team to accomplish tests within two hours.	high (12/14), low (2/14)	high

3. Reporting: useful knowledge gained by residents	high (10/12)	-

**Criteria outcome**		

4. PT&R protocol quality	high	-

5. Testing results - utility	moderate	-

6. Report quality	high	-

**Overall**		

7. Value of overall experience/lessons learned	moderate-high	

During design and planning of PT&R, recruitment of/engagement with residents was judged low, and "conflict burden" among partners high [40,41]. This was the most apparent conflict issue between the university and community partners during the project: neither thought the other was doing enough to engage residents. Results of the other two process criteria evaluated are given below under Household Testing, and under Reporting. PT&R experience by residents was positive, but was human-resource intensive and partner conflicts made CBPR burdensome. Household PT&R was more time-consuming up-front than non-participatory methods because of the need to develop consensus-based testing/reporting protocols from scratch. Despite our use of CARE-type criteria [39] for test selection, reaching consensus on tests was arduous and conflict laden. The most controversial of tests among partners was the suspended mold spore test because the interpretation of results is ambiguous. The university partner wanted to carry out the test to gather exposure data, while the community partners did not want to produce results having uncertain interpretation. Another test - lead in water - was deemed unnecessary by the university partner based on numerous previous studies of City water showing no evidence for concern, the relatively high cost of testing and technical difficulty (residents having to take first and second draw samples - see Table 1). But community partners disagreed strongly because the indicator was of high priority so the test was chosen. Conversely, the university partner liked the colorimetric water testing for simplicity, multiplicity of indicators (including lead) and low cost, but community partners mistrusted it, so it was eliminated after phase I. When consensus could not be reached, the majority vote decided the action; at most the academics were a minority (2/5) in a five-member quorum.

For the neighborhood PM walks, residents' engagement with design and planning was very low and conflict burden was high, but both became moderate during execution. The community-based partners were reluctant to undertake any walks without residents, while the university partner was concerned that too few data would be gathered if we required participation. A compromise was reached that expended reasonable effort to engage residents, and undertook additional walks to gather more data. We attempted to resolve conflicts through dialogue during which partners exchanged views with frankness, and spaces were opened for compromise. Specifically, the conflict over mold testing was resolved by a special partners' meeting. The university partner presented its case for inclusion based on published literature and the precautionary principal, and concerns were shared. A lack of clarity about partners' roles and responsibilities at the outset contributed to conflict, so we tried to clarify them during conflict resolution and revisit them periodically. In hindsight, mistrust and discord may have been fueled by turf conflict over toxics testing - community-based partners may have viewed household testing as their domain - and also by inadequate community engagement capacity - the difficulty recruiting residents of color, and their lack of representation at the partners' table. Notwithstanding, overall experience and lessons learned were judged moderate-high value.

**Figure 1:**
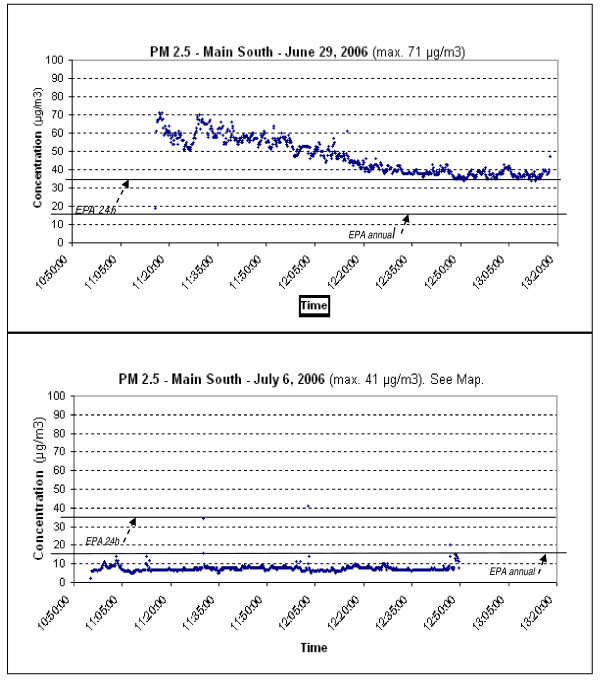
**Time series of PM2.5**. Plot of July 6 corresponds to Figure 2. Reference lines are NAAQS 24-h average standard (35 *μ*g/m^3^) and annual average (15 *μ*g/m^3^). While the sensor tends to overestimate actual levels - so direct comparisons with standards are inappropriate - the data may be a surrogate for actual levels.

**Figure 2:**
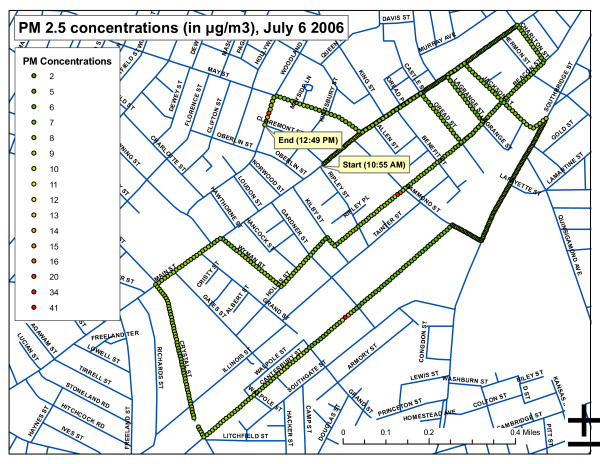
**Map of PM2.5 levels on July 6, 2006 walk**. Data was collected by synchronizing PM meter readings with a GPS unit.

Data were downloaded from the Scout to a laptop computer immediately after the walk during a picnic with residents, and displayed as time series graph of PM as a function of time (Figure 1). The real-time data proved able to stimulate useful dialogue about trends, highs and lows, and implications for respiratory health, especially asthma, a health problem with high prevalence in local children and adults [6,7]. Participants were able to understand more clearly the relationship between PM and asthma since we used the data they helped us collect to talk about their exposure and potential health risks. We related spikes in outdoor PM levels to specific construction-site sources as we walked. Residents and researchers discussed the need to monitor at different times of the day, especially in an effort to capture local sources: Interstate 290 runs on the edge of Main South, and the Container Port is there too. The impact of idling trains was a concern of participants, and is a focus of follow-up monitoring efforts using PT&R. Internet access-permitting, while viewing our data we accessed the City's Summer Street air quality monitoring station and compared them to official hourly PM2.5 levels. While detailed comparison was not possible because our sampling times were much shorter - we had only 1-2 hourly average data points from the network for comparison - we did see differences, and discussed the importance of PT&R to provide street-level data at the height people are exposed. Maps of PM levels were later produced for each sampling day as the final output (Figure 2), and likewise have proved useful for diverse audiences - from high school teens to health scientists - to stimulate a dialogue about temporal and spatial patterns of PM, exposures, who are the most vulnerable, health risks and actions to reduce them.

The household testing protocol was rated high quality, and test results utility was rated moderate. Effectiveness of participatory testing was as follows: 10/14 homes rated high in ability to engage with each test, 4/14 moderate; 12/14 homes undertook the testing in less than the reasonable threshold of two hours. Results of phase I involving eight homes sampled during summer 2007 are given in Additional File 1 and results of phase II with six homes during winter 2008 in Additional File 2. Lead-in-paint touch-swab results were poor proxies of lead-in-dust: in one case (1010) there were 0/6 swab positives and very high levels (over 1000 *μ*g/ft^2^) of lead in sill dust. Lead in floor dust tended to be much lower that lead in sill dust, with one exception (1030, > 6000 *μ*g/ft^2^). Six of the first eight homes tested in summer exceeded at least one USEPA lead standard for dust and/or soil. Only one home tested in winter exceeded the lead-in-dust standard. In phase I, four homes show yard soil above 1000 ppm; the federal standard for outdoor soil is 400 ppm. Our phase I (summer) indoor mold levels averaged 2340 CFU/m^3^. Our PM2.5 levels averaged 13.0 *μ*g/m^3 ^in living rooms and 14.0 *μ*g/m^3 ^in kitchens (n = 14).

The colorimetric test for lead-in-water in phase I did not detect lead, and laboratory lead-in-water testing was also below the detection limit in all second-draw tap water. Very low levels of lead (3.8, 1.4 ppb) were found in first draw of two units, below the EPA action level of 15 ppb. All other water parameter levels were below EPA maximum contaminant levels or guideline values as shown (Additional File 1), except for one bacteria positive (unit 1000). Average indoor mold levels in the first eight units tested in summer months (2300 spores/m^3^) were an order of magnitude higher compared to the six tested in winter (350 spores/m^3^). One home (2030) showed a snapshot ratio indoor level/outdoor level of 1410/36, suggestive of an indoor mold source. The highest level of indoor radon detected was 2.5 *p*Ci/L (unit 1010), a "moderate risk level" below the 4.0 *p*Ci/L EPA action level; homes with 2.0-4.0 *p*Ci/L are advised to mitigate radon.

Additional File 3 shows an excerpt from a report; the quality and information value of the report was judged high by CBPR partners. The results were put in colorful indexed binders and presented in person to participants, along with additional local, state and national resources to assist them (information sheets, agency contacts). The format was revised after phase I, and further revisions are ongoing to simplify the language used to a 6^th^-grade level; this level is recommended based on local socio-demographic data, including Census and anecdotal information. The level of overall positive response to the reporting was high (10/12 respondents). The main comments were that findings should be returned more promptly, within a 2-3 weeks of testing, and further efforts be made to reduce the time required to test. The reporting did become more efficient as the pilot progressed, and we judge that a three-week turnaround is feasible, limited by external laboratory turnaround. Regrettably we did not undertake follow-up interviews to test the adoption of mitigation options, but this will be done for expansion efforts.

## Discussion

CBPR work is complicated and achieving an ideal participatory dynamic is at best illusive, arguably impossible. Partnerships take time to develop and nurture because they are based on trust and the demonstration of mutual benefits for residents, community-based organizations and academic researchers. Given the newness of our partnership and unforeseen conflicts that were hard to resolve, we achieved moderate-high success overall based on process and outcome criteria: methods, test results, reporting, and lessons learned.

Placing our work in the social context of participatory methods/processes literature is informative. Poverty, political exclusion, environmental injustice, poor educational preparation, racism, classism, and homophobia characterize high-minority marginalized communities, and make it difficult to craft CBPR partnerships among academics, community-based organizations and at-risk residents; power inequities are inherent and pronounced. Making the participatory process broadly representative of diverse, disparate interests requires confronting such inequities and opening-up safe spaces for public and private dialogue through which people define who they are, what they want and how they can achieve it: a 'communication for social change' approach [42]. From the parent project [6] we learned that it is necessary to be transparent with aims and actions, and employ horizontal group structures with overlapping responsibilities but clear roles and benefits for each participant. Regular group reflection and structured dialogue mitigate, but may not prevent conflict burden. One of the major challenges of CBPR partnerships is to continuously and dynamically renegotiate the relationships among partners, working in creative ways so that trust, co-ownership and an energizing sense of pride can be built. In this way, participation, roles, and expectations are made more equitable, and actions and experiences more likely to promote social and environmental justice [43]. The above reflections are in-line with over 25 years experience of participatory methods for socio-economic development and poverty reduction: participatory rural appraisal (PRA), applied in Africa and Asia since the 1980s, participatory action research (PAR), rapid rural appraisal (RRA) and participatory action development (PAD) [44]. A useful summary of participatory tools and stakeholder processes can be found in [45].

CBPR is founded on such methods [8-10], and promotes the active involvement of communities in the shaping of research and intervention, as well as implementation and evaluation of research projects [11]. Unlike conventional positivist scientific research CBPR can ask (and may be able to answer) questions about health, environment and poverty that matter most to those most vulnerable [46]. Kurt Lewin, a social psychologist, coined the term *action research *in the 1940s: the marriage of academic research and community interests to effect social change [47]. From our work on the parent project [6] we found that "action" may be viewed as belonging to community advocacy groups, while "research" is the academics' domain. CBPR academics have to strike a delicate balance, making sure their research informs and promotes action for social change without trespassing on the turf of activist and advocacy groups. More nuanced still, the PT&R work revealed that certain types of action-oriented research (like PT&R itself) can also be undermined by turf conflicts among partners. Lewin (as reported by [47]) recognized the fundamental importance of "intergroup relations", as well as the interactions among academic researchers, the subjects of research, and other groups. Any real or perceived competition or turf squabbles among partners undermine goals and forestall the building of trust, pride and cooperation.

CBPR partners must strive to respond to community concerns and build authentic and empowering partnerships. The term *authentic *refers to the degree to which a project reflects not only community concerns - which we did quite well (see Methods/CBPR) - but also how well it literally 'wears the face of the community', with representatives of affected residents as full partners [24,48] - something we did not do well. Schell et al. [49] describe the use of CBPR to understand health disparities and toxics' exposures among Akwesasne Mohawk young adults in upstate New York. Hiring community members as key personnel, involving the local community in research design and implementation, and developing a community education and outreach program all helped build a more equitable partnership. Corburn [50] writes of CBPR lessons learned in Brooklyn's Greenpoint/Williamsburg District, concluding that "street science" and "academic science" can only be integrated if the participatory process combines flexibility with agreed-upon rules of cooperation, and academics work to understand and respect local 'street' rules and norms. While the academics did the latter (and live in the neighborhood themselves), flexible but structured cooperation was illusive. Shephard et al. [51] describe outdoor CBPR led by environmental-justice organization *West Harlem Environmental Action *(WE ACT): outdoor air monitoring, asthma research, training courses for community leaders, and educational forums for local residents. They conclude "to effect meaningful change in the environments and health of communities of color and low-income communities, community-based organizations [CBOs] and leaders must engage the larger public and work in coalition with government agencies, academic institutions, public and private foundations, policymakers, legal experts and local businesses" [[51], p.140]. We suspect that when it is the CBO that approaches the university partner, instead of the other way around (as in our case), engagement between residents and researchers is likely to be more effective, provided the latter embrace CBPR values.

Seifer [52] lays out twelve common features of successful community-university partnerships. While we had most of these, top of the list is "trusting relationships", and this was lacking, as was a spirit of mutual support and mutual benefit, even though all partners were involved in conceiving the work. Israel et al. [53] examined three requisites for CBPR sustainability: 1) sustaining relationships and commitments among the partners involved; 2) sustaining the knowledge, capacity and values generated from the partnership; and 3) sustaining funding, staff, programs, policy changes and the partnership itself. Difficulties with the first of these undermined our efforts. Freeman et al. [15] highlight two challenges to CBPR work for environmental health: 1) building "true partnerships" by ensuring equitable funding and resource allocation among partners; 2) and the alignment of objectives and expectations. Our project did pay close attention to the first, but we did poorly at the second despite regular meetings and adherence to the goals of the proposal. In our PT&R work, like the Healthy Public Housing Initiative (HPHI) work described in [15] tension between action and research was an anticipated source of conflict. But unlike HPHI it was not a source of creative development, rather became a source of persistent conflict. In Yonas et al. [54] the challenge of racism is central. Not having residents of color in the community-university partnership was not a result of racism, rather a naive assumption that community based groups would represent them. There was no intentional exclusion or discrimination, but the lack of racial diversity among the partners could be misconstrued as such, undermining the legitimacy of the group in the eyes of residents, and its effectiveness at engagement and action.

The key CBPR lesson we learned is that future PT&R work should fund the active participation of a few motivated residents of color as representatives of the target population and not rely on community-based groups (who may be predominantly white and middle-class) to try to represent its interests and concerns. Indeed, training new local leaders to be vital human resources who connect the academic researchers to local residents is highly desirable and likely governing of sustainable impact. In ethical terms, having such representatives as funded partners of PT&R, alongside community groups and researchers, also offers the best chance of weighing its benefits and harms.

The PM monitoring walks were an enjoyable activity which residents were able to engage with, and the production of time series (Figure 1) stimulated learning and dialogue. Data showed that there is very large day-to-day variability in PM2.5 and PM10 levels. Norris et al. [55] found that levels of PM2.5 below the current NAAQS annual standard of 15 *μ*g/m^3 ^resulted in a significant increase in emergency department visits for asthma in children. While the June 2006 plot indicates levels of PM2.5 well above the 24-hour standard, we must be careful in our interpretation: there is some uncertainty about the accuracy of nephelometer methods for PM mass concentration measurement [56]. They tend to overestimate PM levels [37] so direct comparison with NAAQS is inappropriate. However, Shendrikar and Steinmentz [57] found such measurements to be a surrogate for regional airborne PM2.5 levels.

While exposures to high lead-in-dust and yard soil are of concern for six of the fourteen homes tested, the lack of standards for mold and PM in the home environment makes interpretation of those data problematic. Comparison with other indoor testing data is useful. Our lead-in-floor dust levels in summer (Additional File 1) show five of the eight homes tested with levels well above the national estimate; the estimate of the geometric mean (GM) of lead in floor dust in U.S. housing for 1998-2000 was 1.1 μg/ft^2 ^[17,58]. In a high-risk houses intervention study, floor dust levels dropped from 14 *μ*g/ft^2 ^(GM) immediately after intervention 4.8 *μ*g/ft^2 ^six years after hazard control [59].

Our summer indoor mold levels averaged 2340 total spores/m^3 ^(Additional File 1). According to limited evidence [60,61] levels over 1000 spores/m^3 ^may be unhealthy and trigger allergic response. In a study of indoor mold levels [62], counts detected indoors at 85 residential structures ranged from 68 to 2307 spores/m^3^. A large proportion of structures had indoor mold levels >500 total spores/m^3 ^- a common action level for remediation when occupants complain of nonspecific adverse health symptoms.

Our PM2.5 levels averaged 13.0 *μ*g/m^3 ^in living rooms and 14.0 *μ*g/m^3 ^in kitchens (n = 14), but averaging time was only 30 minutes. Levels in living rooms and kitchens may be indicative of indoor smoking and/or cooking. In the pilot we did not flag PM sources attributable to personal behavior, but they do need to be considered in follow-up work and require a careful addition to the reporting tool, one that respectfully communicates information about personal behavior and how to modify it (e.g. smoking outdoors, using/maintaining cooking exhaust fans). In a study of regional levels of PM2.5 in Virginia, a sample of 50 homes yielded indoor 24 h averages of 20.2 *μ*g/m^3 ^(SD = 9.9) [63]. In Gothenburg, Sweden, median levels (n = 30) indoors were 8.6 *μ*g/m^3 ^[64].

Report-back is a central part of ongoing work by Communities for a Better Environment and Silent Spring Institute [31,32]. It follows the same CBPR approach that our study used, respecting the rights of study participants to information before, during, and after studies so that they can make informed decisions and take actions to reduce exposure [65,66]. The format of our report (Additional File 3), however, was simpler: it did not include technical graphs used in other studies [67] because our target population on average has a 6^th^-grade literacy level, and during preparatory workshops participants preferred a "less is more" approach.

Childhood lead poisoning prevention has been a longstanding local priority. In 2005, our partnership was instrumental in forming the *Worcester Lead Action Collaborative (WoLAC)*. This multi-stakeholder action group focuses on the priority health risk of childhood lead poisoning, and is dedicated to education and outreach, risk prevention and making homes much more lead-safe through 'abatement'/professional remediation. In 2007, the group won a $3 M grant from HUD for strategic lead 'abatement'/remediation that targets vulnerable populations, and a further $6 M in 2009. Through the Collaborative and the Massachusetts Department of Public Health, we are using our results to advocate for changes to the existing lead testing procedure: the pilot project has provided some evidence for the importance of soil testing as well as dust and paint testing; soil may be an important source of indoor lead. Our pilot project is informing the new holistic orientation of WoLAC as it evolves into a "Healthy Homes-Healthy Communities" Collaborative. Our wider goal of community empowerment is also being met by the creation of a community accessible website - see *Neighborhood STRENGTH.org *- that disseminates results to residents, community groups, public agencies and researchers. Teens in a science class at the University Park Campus School in Main South became partners on neighborhood PM monitoring, and created their own maps of PM levels around the school. Concerned about their health, some changed the way they walk to school, avoiding high PM indicated on Main Street, and have presented findings to peers and a Town Hall meeting.

Research involving people, their personal environment, and factors that directly affect them, creates professional ethical obligations and challenges. Researchers are obliged to inform people about what they are doing, to secure consent, to avoid harm and, when possible, benefit individuals and communities, and importantly to communicate responsibly their findings to those involved [68,69]. In practice, however, these obligations can appear ambiguous or even conflicting, especially when the information developed or its implications are uncertain and/or when people have little capability to respond to or make use of the information; meeting such obligations often appears to pose insuperable difficulties. CBPR/PT&R brings these issues into the open and provides a forum for what may be difficult discussions and for practical work trying to find resolutions. In the pilot, whether or not to make measurements of airborne mold and how to interpret and report those measurements provoked particularly intense discussion. We describe some of our experience with the expectation that it will help inform, though not resolve the discussion of these issues. More broadly, we argue that paying special attention to the researcher-participant relationship, trust-building and participant empowerment, along with the grounding of technical findings in residents' personal experience can be valuable ingredients in comprehensive toxics testing.

*How do results inform household testing practice? *Evaluation of the pilot's processes, outcomes and overall experience show PT&R to be an option worthy of serious consideration, but one with clear advantages and disadvantages. Advantages of PT&R over expert-driven testing include: a) improved researcher-participant communication, cooperation and trust-building; b) education of participants through hands-on training; c) risk communication tailored to personal household contexts; d) capacity building of community partners in technical aspects of environmental testing, and also in community engagement/mobilization centered on household toxics; e) co-ownership between researchers and residents that may improve participant retention, especially in long-term studies. Our National Children's Study for Worcester County project, for example - a partnership led by University of Massachusetts' Medical School - is benefiting from the pilot PT&R experience as we plan household testing and seek to maximize retention through co-ownership. NCS is a 21-year nationwide study of how environmental factors impact child health and development [70]. On the other hand, disadvantages include: a) high human resource costs during design, planning and implementation stages, especially at start-up/piloting - total people-hours, numerous coordination and communication effort (meetings' fatigue), a steep technical learning-curve for many members; b) high transaction costs associated with consensus-driven CBPR, and the need for conflict resolution if conflicts between researchers and community partners become burdensome; c) time to achieve publishable results may be longer; d) limits on the types of test accessible to participant engagement; these are biased towards relatively simple tests with readily interpretable results. Our testing ranged in technical complexity from moderate (bio-aerosol sampler) to low (lead-in-dust wipes). Specifically, we were unable to find affordable real-time gas sensors (e.g. for SO_2_, O_3_, NOx, VOCs/SVOCs, EDCs) sensitive enough to detect ambient home levels.

Our practical experience with the pilot can inform the biomedical ethics debate about balancing the benefits and harms of reporting back personal environmental and biological test results to participants [68,69]. Our partnership agreed that it is unethical not to report-back to residents who both own the information and have the health-based stake in results, but it must be done in ways that are empowering, and this can be difficult and generate disagreement. PT&R offers one approach to coping with these difficulties. It has the potential to educate participants and build capacity among community partners, and is thus empowering and desirable; and it may also point the way to securing outside resources for the community as with the development of the Worcester Lead Action Collaborative. But PT&R may also be disempowering if the burdens to participants being tested and community partners designing and implementing it outweigh the capacity building benefits. Using tests in a PT&R approach which yield results that cannot be interpreted without considerable uncertainty (like airborne mold) presents a right-to-know conundrum: knowing my home had a mold count of 1200 spores/m^3 ^on one occasion is one thing, but what does it mean for my health? Perhaps the uncertainty will worry me more? Our conflict resolution over mold testing shows, however, that the PT&R process provides an opportunity for dialogue and coping with uncertainty even if it does not resolve the right-to-know conundrum. The degree to which information is "actionable" is a function of individual household capacity so the "What action should I take?" section of the report-back (Additional File 3) must be mindful of this fact and emphasize the best affordable, accessible actions.

## Conclusions

Timely reporting back home-toxics' results to residents is ethical, but it must be empowering; not doing so is unethical. Representatives of the target population should be funded partners in CBPR, not simply community-based organizations, while partners need to be mindful of minimizing territoriality and mitigating conflict burden. Equipment manufacturers, researchers, health and housing agencies, and residents should work together to make multi-parameter tests more accessible and empowering; a comprehensive set of tests with PT&R integral may work best. The moderate-high success of the pilot suggests the potential to engender trust and a sense of co-ownership between researchers and residents that would likely improve participant retention and the overall positive impact of environmental health research efforts.

## List of abbreviations

CBPR: community based participatory research; CBO: community based organization; HHP: Healthy Homes Project; CHW: community health worker;

## Competing interests

The authors declare that they have no competing interests.

## Authors' contributions

TD was the academic representative on the ToxicsWatch group and contributed environmental health science and capacity-building knowledge. LR contributed social science, CBPR and local fieldwork and networking expertise. DM and MCC were research assistants who undertook the pilot testing together with our CBO partners, helped finalize report design, and produced reports for interpretation by the partners. OT facilitated meetings and was project manager. RG provided technical advice and assisted with conflict resolution. All authors read and approved the final version of this manuscript.

## Supplementary Material

Additional file 1 **Phase I testing results**. Data show several homes with very lead levels in dust/soil, chronic exposure to which would represent significant lead-poisoning risk, especially for infants who play in soil/floor dust and ingest it, and who grab on to sills. Data above EPA standards for household lead are shown in bold; six of eight homes exceed at least one of them. Other parameters do not indicate exposures of concern, except home 1000 that tested positive for bacteria in tap water. Four homes show yard soil above 1000 ppm. Click here for file

Additional file 2 **Phase II results**. Colorimetric water testing from phase I was discontinued because of a lack of consensus about results accuracy. Data show home 2010 with very high lead levels. Home 2030 data may suggest indoor sources of mold (indoor/outdoor >30). Other parameters do not indicate exposures of concern. Click here for file

Additional file 3 **Example report page**. Each household receives a tailored report about 4-5 pages long, covering all of the chosen indicators, and *ToxicsWatch *explains the results in person. In response to feedback during preparatory workshops with participants, we adopted a "less is more" approach to the design. Such clarity is especially well-suited to environmental justice communities. In hindsight, including web links for more information is a good idea. Click here for file
