# Supplementary material for: Participatory testing and reporting in an environmental-justice community of Worcester, Massachusetts: a pilot project
Source: Environ Health. 2010 Jul 6;9:34. doi: 10.1186/1476-069X-9-34 (PMC2914716; doi:10.1186/1476-069X-9-34)
Supplement: Additional file 1 — Phase I testing results. Data show several homes with very lead levels in dust/soil, chronic exposure to which would represent significant lead-poisoning risk, especially for infants who play in soil/floor dust and ingest it, and who grab on to sills. Data above EPA standards for household lead are shown in bold; six of eight homes exceed at least one of them. Other parameters do not indicate exposures of concern, except home 1000 that tested positive for bacteria in tap water. Four homes show yard soil above 1000 ppm. [file 1476-069X-9-34-S1.DOC]

**Additional File 1. Phase I testing results.** Data show several homes with very lead levels in dust/soil, chronic exposure to which would represent significant lead-poisoning risk, especially for infants who play in soil/floor dust and ingest it, and who grab on to sills. Data above EPA standards for household lead are shown in bold; six of eight homes exceed at least one of them. Other parameters do not indicate exposures of concern, except home 1000 that tested positive for bacteria in tap water. Four homes show yard soil above 1000 ppm.

| **Home ID** | **date tested** | **time of day** | **basement radon** (*p*Ci/L) | **mold indoors** (total spores/m^3^) | **lead in wall paint** (no.+ve/total no) | **lead in floor dust^1^** (µg/ft^2^) - 1st | **lead in floor dust^1^** (µg/ft^2^) - 2nd | **lead in floor dust^1^** (µg/ft^2^) - 3rd | **lead in sill dust^2^** (µg/ft^2^) - 1st | **lead in sill dust^2^** (µg/ft^2^) - 2nd | **lead in sill dust^2^** (µg/ft^2^) - 3rd | **tap water 1st draw** (ppb) | **tap water 2nd draw** (ppb) | **lead in tap** (yes/no) | **pesticides** yes/no | **nitrate** (ppm) | **nitrite** (ppm) | **pH** | **hardness** (ppm) | **chlorine** (ppm) | **bacteria** (yes/no) | **PM2.5 living room** – mean^4^ (µg/m^3^) | **PM2.5 kitchen** – mean^4^ (µg/m^3^) | **lead in yard soil^3^** (ppm) - 1st composite | **lead in yard soil^3^** (ppm) - 2nd composite |
| --- | --- | --- | --- | --- | --- | --- | --- | --- | --- | --- | --- | --- | --- | --- | --- | --- | --- | --- | --- | --- | --- | --- | --- | --- | --- |
| 1000 | 6/26/07 | 16:30 | 0.9 | 2510 | 0/6 | < | < | < | n/a | < | < | < | < | n | n | < | < | 10 | 0 | < | **y** | 14 | 16 | - | 119 |
| 1010 | 7/16/07 | 9:00 | 2.5 | 3820 | 0/6 | 12.6 | < | n/a | **1946** | **3220** | n/a | 3.8 | < | n | n | < | < | 10 | 50 | < | n | 5 | 5 | **1875** | 365 |
| 1020 | 7/24/07 | 15:30 | 1.0 | 2070 | 1/6 | 19.2 | 26.5 | 13.7 | 16.0 | 112.0 | **311.0** | < | < | n | n | < | < | 7 | 100 | < | n | 3 | 2 | **860** | **1410** |
| 1030 | 6/29/07 | 14:00 | 0.4 | 2670 | 5/6 | < | 35.1 | **6190** | 202.1 | 43.0 | 184.0 | < | < | n | n | < | < | 6.5 | 50 | < | n | 2 | 2 | 83 | 161 |
| 1040 | 6/24/07 | 12:30 | 1.2 | 880 | 1/6 | < | < | < | < | < | < | < | < | n | n | < | < | 8.5 | < | 2 | n | 2 | 2 | 250 | - |
| 1050 | 6/25/07 | 10:30 | 0.1 | 702 | 3/6 | 37.5 | 23.6 | < | **397.3** | **636.0** | **1810** | < | < | n | n | < | < | 6 | 120 | < | n | 7 | 7 | **2637** | **1380** |
| 1060 | 7/18/07 | 18:30 | n/a | 3780 | 0/6 | 39.0 | **96.8** | < | 14.4 | 15.8 | 33.6 | 1.4 | < | n | n | 0.25 | 0.1 | 8 | 0 | < | n | 39 | 40 | **1035** | - |
| 1070 | 7/26/07 | 19:30 | 1.0 | 2290 | n/a | < | < | < | < | < | < | < | < | n | n | < | < | 8 | 75 | < | n | 17 | 16 | **617** | 70 |

< less than detection limit (safe by USEPA standards); y yes; n no; - no sample taken; *KT wall, office baseboard, BR mirror, BA wall, HW wall next to BR.

^1^ lead-in-floor-dust USEPA standard ≤ 40 *µ*g/ft^2^; ^2^ lead-in-sill-dust USEPA standard ≤ 250 *µ*g/ft^2^; ^3^ lead-in-bare soil/children’s play areas USEPA standard ≤ 400 ppm (Federal Register 2001); ^4^ 30-minute mean.
