# Supplementary material for: Participatory testing and reporting in an environmental-justice community of Worcester, Massachusetts: a pilot project
Source: Environ Health. 2010 Jul 6;9:34. doi: 10.1186/1476-069X-9-34 (PMC2914716; doi:10.1186/1476-069X-9-34)
Supplement: Additional file 2 — Phase II results. Colorimetric water testing from phase I was discontinued because of a lack of consensus about results accuracy. Data show home 2010 with very high lead levels. Home 2030 data may suggest indoor sources of mold (indoor/outdoor >30). Other parameters do not indicate exposures of concern. [file 1476-069X-9-34-S2.DOC]

**Additional File 2. Phase II results.** Colorimetric water testing from phase I was discontinued because of a lack of consensus about results accuracy. Data show home 2010 with very high lead levels. Home 2030 data may suggest indoor sources of mold (indoor/outdoor >30). Other parameters do not indicate exposures of concern.

| **Home ID** | **date tested** | **time of day** | **basement radon** (*p*Ci/L) | **mold indoors** (total spores/m^3^) | **location** | **mold outdoors** (total spores/m^3^) | **location** | **lead in wall paint** (no.+ve/total no) | **location** | **lead in floor dust^1^** (µg/ft^2^) - 1st | **location** | **lead in floor dust^1^** (µg/ft^2^) - 2nd | **location** | **lead in floor dust^1^** (µg/ft^2^) - 3rd | **location** | **lead in sill dust^2^** (µg/ft^2^) - 1st | **location** | **lead in sill dust^2^** (µg/ft^2^) - 2nd | **location** | **lead in sill dust^2^** (µg/ft^2^) - 3rd | **location** | **tap water 1st draw** (ppb) | **tap water 2nd draw** (ppb) | **PM2.5 living room** – mean^3^ (µg/m^3^) | **PM2.5 kitchen** – mean^3^ (µg/m^3^) |
| --- | --- | --- | --- | --- | --- | --- | --- | --- | --- | --- | --- | --- | --- | --- | --- | --- | --- | --- | --- | --- | --- | --- | --- | --- | --- |
| 2000 | 2/01/08 | 9:30 | 2.3 | 150 | KT | 170 | HW | 1/6 | LR | < | L R | < | BR | < | KT | < | LR | < | BR | 17 | KT | 36.7 | 1.7 | 9 | 8 |
| 2010 | 2/01/08 | 16:45 | 0.6 | 30 | HW | 30 | EN | 1/6 | BR | < | L R | < | BR | < | HW | **15000** | LR | **2100** | BR | 45.0 | DR | 1.1 | <1 | 3 | 4 |
| 2020 | 2/06/08 | 18:50 | n/a | 120 | KT | 360 | EN | 0/6 | n/a | < | LR | < | BR | < | KT | < | LR | < | BR | n/a | n/a | - | - | 5 | 6 |
| 2030 | 3/03/08 | 10:30 | n/a | **1410** | LR | **36** | WW | 5/6 | * | 9.8 | LR | < | BR | 27.0 | KT | 19.0 | LR | < | BR | 13.0 | KT | 2.7 | 1 | 59 | 72 |
| 2040 | 3/03/08 | 14:50 | 0.1 | 350 | LR | 36 | WW | 1/6 | BR | < | LR | < | KT | n/a | n/a | 18.0 | LR | 21.0 | BA | n/a | n/a | <1 | <1 | 9 | 9 |
| 2050 | 3/19/08 | 15:30 | n/a | 30 | LR | 10 | PO | 1/6 | KT | < | DR | < | KT | 0* | BR | < | DR | < | KT | < | BR | 15 | <1 | 7 | 7 |

^1^ lead-in-floor-dust USEPA standard ≤ 40 *µ*g/ft^2^; ^2^ lead-in-sill-dust USEPA standard ≤ 250 *µ*g/ft^2^; ^3^ 30-minute mean.

**Key**: LR - living room, BR - bedroom, DR - dining room, KT - kitchen, w.sill - window sill, HW - hallway, EN - entrance, PO - porch, WW - walkway, BA – bathroom; *KT wall, office baseboard, BR mirror, BA wall, HW wall next to BR; < below detection limit; - resident did not take sample.

# 
