# Supplementary material for: Participatory testing and reporting in an environmental-justice community of Worcester, Massachusetts: a pilot project
Source: Environ Health. 2010 Jul 6;9:34. doi: 10.1186/1476-069X-9-34 (PMC2914716; doi:10.1186/1476-069X-9-34)
Supplement: Additional file 3 — Example report page. Each household receives a tailored report about 4-5 pages long, covering all of the chosen indicators, and ToxicsWatch explains the results in person. In response to feedback during preparatory workshops with participants, we adopted a "less is more" approach to the design. Such clarity is especially well-suited to environmental justice communities. In hindsight, including web links for more information is a good idea. [file 1476-069X-9-34-S3.DOC]

**Additional File 3. Example report page.** Each household receives a tailored report about 4-5 pages long, covering all of the chosen indicators, and *ToxicsWatch* explains the results in person. In response to feedback during preparatory workshops with participants, we adopted a “less is more” approach to the design. Such clarity is especially well-suited to environmental justice communities. In hindsight, including web links for more information is a good idea.

| ***My Home Test Results*** | | | |
| --- | --- | --- | --- |
| **Pollutant** | **Why do we test for Lead?**  **Lead is a metal that was used in paint, plumbing, gasoline, and other products. Even though many of these uses are now banned, lead remains in our homes and environment. Lead can hurt the brain, kidneys, and nervous systems of children.** | | |
|  | **My Result** | **What does my result mean?** | **What action should I take?** |
| **Lead in water**  [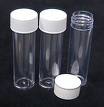](http://images.google.com/imgres?imgurl=http://www.lifestylestore.com/images/5033.jpg&imgrefurl=http://www.lifestylestore.com/ls_vials%26goodies.html&h=216&w=209&sz=10&hl=en&start=3&um=1&tbnid=PfkO2HzaHl9SpM:&tbnh=107&tbnw=104&prev=/images%3Fq%3Dplastic%2Bvials%26svnum%3D10%26um%3D1%26hl%3Den) | First Draw and Purged Line   | X X |  |  |  | | --- | --- | --- | --- | | Less than 1 | 1 to 10 | 10 to 30 | More than 30 |   Lead in water is measured in parts per billion (ppb), which tells us the amount of lead found in a given amount of water. Even small amounts of lead can be harmful. | The lab did not find any lead in your water. | No action required. |
| **Lead-in-dust**  **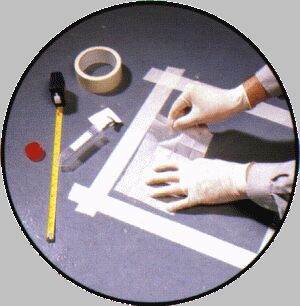** | Living Room and Kitchen Floor   | **X X** |  |  | | --- | --- | --- | | Less than 10 | 10 to 40 | More than 40 |   Living Room Windowsill   |  | 18 |  | | --- | --- | --- | | Less than 10 | 10 to 250 | More than 250 |   Bathroom Windowsill   |  | 21 |  | | --- | --- | --- | | Less than 10 | 10 to 250 | More than 250 |   Lead in dust is measured as micrograms per square foot (µg/ft^2^). | The US Environmental Protection Agency says lead-in-dust is hazardous when the level reaches 40 micrograms per square foot on floors and 250 micrograms per square foot on inside window sills.  All of the dust samples from your home were below these limits. | This test indicates there is at least some lead paint in your home. However, the levels of lead in your dust were well below EPA hazard levels.  If you still have concerns, read the attached lead information. This is in the purple folder. You can also read the recommendations in the lead in paint section below. |

| ***My Home Test Results*** | | | |
| --- | --- | --- | --- |
| **Mold/Fungus/ Moisture**  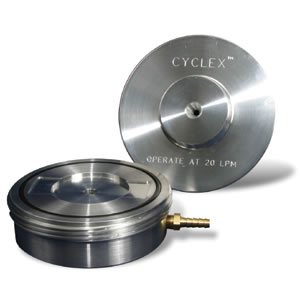 | **Why do we test for Mold?**  **Exposure to mold can lead to asthma attacks, allergic reactions, rashes, headaches, tiredness, sneezing, and watery eyes. Mold grows on wet or damp surfaces, so moisture control is the key to mold control.** | | |
|  | **My Results** | **What does my result mean?** | **What action should I take?** |
|  | Inside Living Room:  **350** spores per cubic meter (spores/m^3^)  Outside Walkway:  **36** spores per cubic meter (spores/m^3^) | We included the test for mold because exposure to mold can contribute to health problems such as asthma. However, the results are difficult to interpret without more information because scientists disagree about what levels of mold represent a health risk. Some studies suggest that mold spore levels of as low as 1000 spores/m^3^ may be risky. Others believe spore counts 10 times higher inside than outside indicate an indoor mold problem.  Other factors are also involved. For example, some spores are more risky than other and spores that come from inside your home may be more risky than spores that come from outside your home. Your inside spore count is about 10 times higher than the outside mold count. The spore that is most present in your home is *Aspergillus*, which is found in water damaged buildings. | Your mold spore results are difficult to interpret because no standard exists.  Your result is below 1000 spores/m^3^ which most scientists consider safe. However, your inside spore count is about 10 times higher than the outside mold count, so there may be some water damage.  At this time, the best thing to do is to monitor any moisture and water damage in your apartment. If mold growth appears, you should speak with your landlord about eliminating the moisture source.  You should also read the enclosed materials on moisture and mold. These materials are in the green folder. |
